# LC-ESI-MS/MS Phenolic Profile of* Volutaria lippii* (L.) Cass. Extracts and Evaluation of Their* In Vitro* Antioxidant, Antiacetylcholinesterase, Antidiabetic, and Antibacterial Activities

**DOI:** 10.1155/2019/9814537

**Published:** 2019-06-24

**Authors:** Hichem Ben Salah, Slim Smaoui, Raed Abdennabi, Noureddine Allouche

**Affiliations:** ^1^Laboratory of Organic Chemistry LR17ES08 (Natural Substances Team), University of Sfax, Faculty of Sciences of Sfax, Sfax, Tunisia; ^2^Laboratory of Microorganisms and Biomolecules of the Center of Biotechnology of Sfax, Road of Sidi Mansour Km 6, Sfax 3018, Tunisia; ^3^Laboratory of Plant Biotechnology Applied to Crop Improvement, Faculty of Science, Sfax University, Sfax, Tunisia

## Abstract

*Volutaria lippii *(L.) Cass., an indigenous perennial herb from the Tunisian flora, belongs to the medicinally important genus* Volutaria *Cass. (*Asteraceae*) which comprises eighteen species widely distributed in the Irano-Turanian and Mediterranean Basin. In this study, five different extracts from Tunisian* Volutaria lippii* (L.) Cass. were evaluated for their* in vitro* antioxidant, antiacetylcholinesterase, antidiabetic, and antibacterial activities as well as for their total phenolic and flavonoid contents. The results indicated that the ethyl acetate and aqueous fractions have the highest levels in phenolic and flavonoid contents and showed remarkable antioxidant activities using DPPH (IC_50_= 11.50±0.57 and 28.81±1.35*μ*g/mL, respectively), total antioxidant capacity (105.21±0.01 and 98.77±0.02 mg vitamin E/g extract, respectively), and reducing power (EC_50_= 55.40±2.00 and 66.65±1.40 *μ*g/mL, respectively) methods. Furthermore, they exhibited noticeable antiacetylcholinesterase and antidiabetic activities and a moderate antibacterial effect when compared to that of standards. Principal component analysis allowed highlighting the ethyl acetate extract for its interesting acetylcholinesterase enzyme (AChE) and alpha-amylase activities and the aqueous fraction for its remarkably antibacterial activity, and their richness in phytochemical content. Interestingly, the LC-ESI-MS/MS analyses of both fractions allowed the identification of ten phenolic acids and eight flavonoids. The 3-*O*-caffeoylquinic and 3,4-di-*O*-caffeoylquinic acids constituted the most abundant components in the two fractions. Taken together, these findings demonstrated, for the first time, that* Volutaria lippii* (L.) Cass. is a potential source of biological active compounds which could be used in a wide range of fields, namely, nutrition and complementary pharmacological drug.

## 1. Introduction

The Tunisian flora has a wide plant varieties used not only in folk medicine, but also in pharmaceutical, cosmetic, and food technologies [1]. These plants represent an excellent reservoir for extracting and identifying bioactive phytochemicals, which exerted a beneficial effect on human healthiness [2] and had a preventive role against cancer and chronic diseases. Many of these natural compounds, such as polyphenols, flavonoids, and phenolic acids, are known for their various pharmacological activities including antioxidant, antimicrobial, antidiabetic, anti-inflammatory, anticancer, and anti-Alzheimer effects [3, 4].

*Volutaria lippii* (L.) Cass. ex Maire (syn* Centaurea lippii* L.,* Volutarella lippii* (L.) Cass.,* Amberboa lippii* (L.) DC.) is one of the Tunisian plants that belong to the genus* Volutaria* Cass., tribe Cardueae, subtribe Centaureinae of the* Asteraceae* (*Compositae*) [5]. The genus* Volutaria* comprises approximately eighteen species growing in semiarid to arid zones and widely distributed in the Irano-Turanian and Mediterranean areas [6]. Pharmacological and phytochemical studies on several* Volutaria* species have reported that these plants are rich in sesquiterpene lactones and flavonoids which possess various biological activities [7, 8]. Previous study reported the isolation of one sesquiterpene lactone identified as cnicin and three flavonoids, identified as nicotiflorin, isovitexin and isoquercitrin, from the n-butanol extract of* V. lippii* [9]. Other works released on* V. lippii* were conducted to the isolation of two sesquiterpene lactones identified as amberboin and grosshemin [10, 11].

The aim of actual study was to evaluate the phytochemical screening (flavonoid and total phenolic contents) and* in vitro* antioxidant, antiacetylcholinesterase, antidiabetic, and antibacterial potentials of* V. lippii*. Moreover, the LC-ESI-MS/MS technic was employed for qualitative and quantitative analyses of phytochemicals in ethyl acetate and aqueous fractions. This study was the first report depicting chemical profile and biological property evaluations of* V. lippii* extracts.

## 2. Material and Methods

### 2.1. Plant Material

Aerial flowering parts of* V. lippii *were collected in April 2014 from Sfax in the central east of Tunisia (34°44′26.02′′N, 10°45′37.01′′E). A voucher specimen (Number LCSN 118) was deposited in the Herbarium Laboratory of Organic Chemistry (Natural Substances Team), Faculty of Sciences, Sfax University, Tunisia.

### 2.2. Chemicals and Reagents

2,2-Diphenyl-1-picrylhydrazyl (DPPH), butylated hydroxytoluene (BHT), vitamin E (*α*-tocopherol), gallic acid, quercetin, potassium ferricyanide, ferric chloride, ammonium molybdate, acetylcholinesterase (AChE), tacrine, *α*-amylase, acarbose, penicillin, Folin-Ciocalteu phenol reagent, and HPLC grade reagents: quinic acid, caffeic acid, protocatechuic acid, 1,3-di-*O*-caffeoylquinic acid, 3,4-di-*O*-caffeoylquinic acid, 4,5-di-*O*-caffeoylquinic acid, apigenin-7-*O*-glucoside, cirsilineol, and acacetin were purchased from Merck (Sigma-Aldrich, Steinheim, Germany).

### 2.3. Extraction Procedure

Air-dried and powdered aerial part (300 g) of* V. lippii *was extracted by maceration with 80% aqueous-ethanol for 24 hours three times at room temperature with regular stirring. The resulting extracts were collected, filtered, and concentrated under vacuum. The dried crude extract was solubilized in 500 mL of distilled water for fractionation. The aqueous solution was further partitioned successively with hexane, dichloromethane, ethyl acetate, and n*-*butanol. The hexane, dichloromethane, ethyl acetate, n-butanol, and the final aqueous fractions were filtered and evaporated to dryness under vacuum.

### 2.4. Determination of Total Phenolic Content

The total phenolic content in* V. lippii *extracts was estimated by Folin-Ciocalteu method according to Chen et al. [12]. To 100 *μ*L of diluted sample extract, 2mL of Na_2_CO_3_ aqueous solution (2%) and 100 *μ*L of 50% Folin-Ciocalteu reagent were added. The final mixture was incubated for 30 minutes in the dark. The absorbance of each sample was read at 750 nm with a Shimadzu UV/Vis spectrophotometer (Jenway 6320D). The results are expressed in gallic acid equivalents (mg GAE/g extract).

### 2.5. Determination of Total Flavonoid Content

The total flavonoid content in extracts was determined by the method described previously by Djeridane et al. [13] and expressed as quercetin equivalents (mg QE/g extract). Briefly, 1mL of each diluted* V. lippii *fraction was added to 1mL of AlCl_3_ methanolic solution (2%). After 15 min incubation at room temperature, the absorbance of the obtained mixture was measured at 430 nm.

### 2.6. DPPH Radical Scavenging Assay

The DPPH radical scavenging activity of different fractions of* V. lippii* was evaluated following the procedure described by Les et al. [14]. IC_50_ value of each fraction, i.e., concentration of sample necessary to decrease the initial DPPH concentration by 50%, is a parameter widely used to assess the antioxidant activity. Briefly, 1.5 mL of DPPH solution (10^−4^ M, in 95% Ethanol) was added to 1.5 mL of each* V. lippii *fraction at various concentrations (0.01-1 mg/mL). The final concentrations in the reaction's mixture were 0.005-0.5 mg/mL. Each mixture was shaken and allowed in the dark for 30 min at room temperature. The blank was prepared as above without any extract and the BHT was used as positive control. The percentage of inhibition (PI (%)) was determined spectrophotometrically by monitoring the decrease in absorbance at 517 nm against a blank. The PI was calculated using the following equation:

(1) $$\begin{array}{c} PI\left(\;\%\;\right)=\left[\;\frac{A_{\text{blank}}\text{ – }A_{\text{sample}}}{{\text{A}}_{\text{blank}}}\;\right]\times 100 \end{array}$$

where A_blank_ is the absorbance of the blank and A_sample_ is the absorbance of the test sample.

The calibration curve for scavenging percentage against extract concentration was plotted and the IC_50_ (half maximal inhibitory concentration) value of each sample was established.

### 2.7. Total Antioxidant Capacity Assay (TAC)

The total antioxidant capacity (TAC) of all fractions of* V. lippii *was spectrophotometrically assessed by the method of Prieto et al. [15]. A 0.1 mL of each* V. lippii *fraction (1 mg/mL) was mixed with 1 mL of reagent solution (28 mM sodium phosphate, 0.6 M sulfuric acid and 4 mM ammonium molybdate). The mixtures were incubated for 90 min in a boiling water bath at 95°C. After cooling the samples, the absorbance was determined at 695 nm. The antioxidant capacity was expressed as equivalents of vitamin E (*μ*g/g of extract).

### 2.8. Reducing Power Assay

The reducing power of all fractions was evaluated using the procedure of Yildirim et al. [16], and BHT was used as positive control. A 1 mL of different concentrations of each* V. lippii *sample (5, 10, 25, 50,100 *μ*g/mL) was mixed with 2.5 mL of 1% potassium ferricyanide and 2.5 mL of sodium phosphate buffer (0.2 M, pH= 6.6). The mixture was incubated for 20 min at 50°C and then 2.5 mL of 10% trichloroacetic acid were added and centrifuged for 10 minutes. 2.5 mL of the supernatant were mixed with 0.5 mL of ferric chloride solution (0.1%) and 2.5 mL of distilled water. The absorbance of the mixture was measured at 700 nm.

### 2.9. Determination of Acetylcholinesterase (AChE) Inhibitory Activity

The AChE inhibitory activity was performed according to the colorimetric method described by Ellman et al. [17], with modifications. Briefly, 125 *μ*L of DTNB (3 mM), 50 *μ*L of sodium phosphate buffer (pH 8.0), 25 *μ*L of AChE (0.5 U/mL), and 25 *μ*L of each* V. lippii *extract dissolved in DMSO were added in a 96-well microplate and incubated for 15 min at 25°C. The reaction was then initiated by the addition of 25 *μ*L of acetylthiocholine iodide (ATCI) and the hydrolysis of acetylthiocholine iodide was controlled by the formation of the yellow 5-thio-2-nitrobenzoate anion as a result of the reaction of thiocholine with DTNB. Plant fractions were tested for AChE inhibitory activity at the following concentrations 25, 50, 125, 250, and 500 *μ*g/mL. The final concentrations in the reaction's mixture were 2.5, 5, 12.5, 25, and 50 *μ*g/ mL [14]. A reaction mixture containing all the components except the* V. lippii* extract was used as control. Tacrine was used as positive control. The absorbance was then read three times with 3 min intervals at 405 nm by a CERES UV 900C microplate reader (Bio-Tek Instrument, USA). Any increase in absorbance due to the spontaneous hydrolysis of the substrate was revised by subtracting the absorbance before appending the enzyme. The percentage inhibition was calculated as follows:

(2) $$\begin{array}{c} PI\left(\;\%\;\right)=\left[\;\frac{A_{\text{control}}\text{ – }A_{\text{sample}}}{{\text{A}}_{\text{control}}}\;\right]\times 100 \end{array}$$

where A_control_ is the absorbance of the control and A_sample_ is the absorbance of the test sample.

Extract concentration providing 50% inhibition (IC_50_) was obtained by plotting the percentage inhibition against extract concentration.

### 2.10. Determination of *α*-Amylase Activity* In Vitro*

The* in vitroα*-amylase inhibitory assay of all fractions was performed by the previous method described by Gella et al. [18]. The enzyme *α*-amylase solution was made by blending 3.246 mg of alpha amylase (EC 3.2.1.1) in 100 mL of phosphate buffer (40 mM, pH 6.9). The assays were conducted by mixing 30*μ*L of alpha-amylase solution, 120*μ*L of E-PNPG7, and 60*μ*L of each* V. lippii* fraction in the concentration range 25, 50, and 100 *μ*g /mL. The final concentrations in the reaction's mixture were 7.14, 14.28, and 28.57 *μ*g/mL. The positive control (acarbose) was prepared by dissolving 50 mg in 50 mL of phosphate buffer and diluted to get different concentrations 10, 20, and 40 *μ*g/mL. The final concentrations in the reaction's mixture were 2.85, 5.71, and 11.42 *μ*g/mL. The mixture was incubated for 8 min at 37°C. The absorbance was read at 405 nm and control reaction was carried out without the* V. lippii *fractions. Percentage inhibition (PI) was calculated by the following expression:

(3) $$\begin{array}{c} PI\left(\;\%\;\right)=\left[\;\frac{A_{\text{control}}\text{ – }A_{\text{sample}}}{{\text{A}}_{\text{control}}}\;\right]\times 100 \end{array}$$

where A_control_ is the absorbance of the control and A_sample_ is the absorbance of the test sample.

### 2.11. Antimicrobial Activity Assay

The* V. lippii *fractions were assessed against a five bacterial strains: Gram-negative:* Salmonella enterica *(CIP 8039) and* Escherichia coli* (ATCC 8739), Gram-positive:* Staphylococcus aureus *(ATCC 6538),* Bacillus thuringiensis,* and* Enterococcus faecalis *(ATCC 29212). Bacteria not obtained from an ATCC collection were acquired from the Microbiology Department, Faculty of Science, University of Sfax (Tunisia). Bacterial strains were cultured in Mueller-Hinton agar (MHA) for 24 h at 37°C.

The disc diffusion method was employed for the determination of antibacterial activities of* V. lippii *fractions according to the method described by Berghe and Vlietinck [19]. Antibacterial activities were evaluated by measuring the diameters of the inhibition zones against the test organisms and compared to penicillin (10 *μ*g per disk) as the positive control. Tests were carried out in triplicate.

### 2.12. Chromatographic Conditions and Apparatus

HPLC procedures chromatographic separation was performed on Aquasil C18 (Thermo Electron, Dreieich, Germany) column (150 mm × 3 mm, 3 *μ*m particle size). The solvents used were (A) 0.1% formic acid in water and (B) 0.1% formic acid in methanol. The elution gradient established was 10-100% B, 0-45 min; 100% B, 45-55 min and reequilibration duration was 5 min between individual runs. The flow rate of the mobile phase was 0.4 mL/min, the injection volume was 5 *μ*L, and the column temperature was maintained at 40°C. Phenolics present in the fractions were characterized according to their retention times, UV and mass spectra compared with commercial standards when available. The quantification of phenolics was determined based on DAD results, using 280 nm for the phenolic acids and 320 and 370 nm for flavonoids.

The LC-ESI-MS/MS analysis was carried out using a LCMS-8030 triple quadrupole mass spectrometer (Shimadzu, Kyoto, Japan) equipped with an electrospray ionization (ESI). The mass spectrometer was operated in negative ion mode with a nebulizing gas flow of 1.5 L/min, a dry gas flow rate of 12 L/min, a block source temperature of 400°C, a DL (dissolving line) temperature of 250°C, the full scan spectra from 50 to 2000 Da, and the negative ionization mode source voltage-4500 V.

### 2.13. Statistical Analyses

Each calculation was accomplished using SPSS software (version 19.0; SPSS Inc., Chicago, IL, USA). All experiments were carried out as means ± standard deviations of three replicates. Analysis of variance (ANOVA) followed by Tukey's post hoc test were used to establish the differences between means of various groups. The level of significance was fixed at P<0.05. The principal component analysis (PCA) was applied to separate ethyl acetate and water extracts according to all the parameters investigated without any rotation. The biplot type was correlation biplot, the PCA type was Pearson (n), and the coefficient was automatic. The PCA plots and the Pearson correlation matrix were achieved using XLSTAT software for Windows (v.2014.1.08, Add in soft, New York, USA).

## 3. Results and Discussion

### 3.1. Total Phenolic and Flavonoid Contents

The total phenolic (TP) and total flavonoid (TF) contents of different studied fractions from* V. lippii* were determined. As can be seen in Table 1, the levels of phenolic compounds varied significantly (p<0.05) depending on the influence of solvent polarity [20] and have been found to be rich in all fractions except the hexane one. The ethyl acetate fraction showed the highest (p<0.05) amount of phenolic compounds (65.22±0.03 mg GAE/g) followed by the n-butanol (38.83±0.07 mg GAE/g), the aqueous (35.04±0.05 mg GAE/g), the dichloromethane (24.13±0.04 mg GA /g), and the hexane (7.46±0.23 mg GAE/g) fractions.

With regard to the TF, the highest (p<0.05) value was also found in the ethyl acetate fraction (12.50±0.04 mg QE/g), whereas the lower (p<0.05) one was observed in the hexane fraction (2.14±0.05 mg QE/g). These findings were in agreement with those revealed by Karamenderes et al. (2007) for eight different species of* Centaurea *(synonym of* Volutaria*) [21].

### 3.2. Antioxidant Activity

Several studies were devoted to finding natural antioxidants such as phenolic compounds and flavonoids, which are principally responsible for the antioxidant properties of plant [22, 23]. The antioxidant capacity of different plant extracts cannot be evaluated by a single testing method due to the complex nature of phytochemicals [24]. For this reason, three complementary* in vitro* chemical assays, in terms of DPPH radical scavenging, reducing power, and total antioxidant capacity, were applied in order to screen the potential antioxidant properties of* V. lippii* extracts.

#### 3.2.1. DPPH Radical Scavenging Assay

Antioxidant activity in food can be expressed in terms of radical scavenging ability using free radicals. DPPH assay is extensively used to determine the antioxidant property of many plant extracts [25, 26]. It is well known that free radicals have an important role in the autoxidation of unsaturated lipids in foodstuffs and in oxidative cell damage in the human organism resulting in a variety of pathological diseases [27]. Antioxidants can intercept the chain autoxidation of lipids and donate hydrogen to free radicals, particularly to the lipid peroxides radicals, thereby forming stable free radicals, which do not initiate or propagate further lipid oxidation [28]. The DPPH scavenging activity of* V. lippii *fractions, expressed as IC_50_ (*μ*g/mL), were illustrated in Table 1. Obtained results demonstrated that IC_50_ of different fractions ranged from 11.50 ±0.57 to 43.77 ±2.09 *μ*g/mL, indicating their antioxidant potentials compared with the standard BHT (13.00 ±0.57*μ*g/mL). The order of DPPH scavenging ability of fractions and BHT was as follows: ethyl acetate (11.50 ±0.57*μ*g/mL)> BHT (13.00 ±0.57*μ*g/mL)> aqueous (28.81 ±1.35 *μ*g/mL)> dichloromethane (34.25 ±1.69*μ*g/mL)> n-butanol (43.77 ±2.09 *μ*g/mL). The hexane fraction was found inactive.

These results proposed that the tested fractions, especially ethyl acetate and aqueous ones, have a good capability to donate electrons to reactive free radicals converting them into more stable forms [22]. The raised free radical scavenging activity of* V. lippii* is in favour of the implication of phenolic compounds which have been quantified in all its fractions (Table 1).

#### 3.2.2. Total Antioxidant Capacity Assay (TAC)

CAT method offers a broader view of the antioxidant potential of plant extracts and expresses different aspects of antioxidant action [25]. The results depicted in Table 1 showed that the ethyl acetate fraction has the highest (P<0.05) antioxidant capacity (105.21±0.01mg vitamin E/ g extract) that can be related to its high levels in TP and TF. The TAC of* V. lippii* extracts was found to increase in the following order: hexane < dichloromethane < n-butanol < aqueous < ethyl acetate. This result is in good accordance with the phenolic content variation in all fractions.

#### 3.2.3. Reducing Power Assay (FRAP)

The reducing powers of testing fractions and BHT were also determined (Table 1). In this test, all fractions presented dose-dependent activity whose results are lower than that of BHT (EC_50_= 43.35±0.95 *μ*g/mL). In fact, reducing power of* V. lippii *fractions increased and was well correlated when concentration increased. The reducing power was expressed as effective concentration EC_50_ at which the absorbance is 0.5. As indicated in Table 1, the results revealed that the ethyl acetate fraction exhibited the highest (P<0.05) activity (EC_50_= 55.40±2.00 *μ*g/mL), followed by aqueous (EC_50_= 66.65±1.40 *μ*g/mL), n-butanol (EC_50_= 150.0±3.12 *μ*g/mL), dichloromethane (EC_50_= 216.66±2.49 *μ*g/mL), and hexane (EC_50_= 383.33±2.85 *μ*g/mL) fractions.

These reducing properties are often due to the presence of reductones capable of exerting an antioxidant effect by breaking the free radicals chain and by donating a hydrogen atom. It was reported that reductones respond to various precursors of peroxides and therefore prevent their generation [29].

The results obtained with the three antioxidant activity tests revealed that the different plant extracts contained a considerable amount of antioxidant components. Differences in solvent polarities and thus different extractability of the antioxidant components may explain the differences in the antioxidant activity of* V. lippii* extracts. Both ethyl acetate and aqueous fractions have the strongest antioxidant effects that are related to their high levels of phenolic and flavonoid contents (Table 1). Thus,* V. lippii* extracts, especially ethyl acetate, could be used as natural antioxidant agents.

### 3.3. Acetylcholinesterase Enzyme (AChE) Inhibitory Activity

The results related to the AChE inhibitory activity of* V. lippii *extracts are given in Table 2. Only aqueous extract gave a strong AChE inhibition (P<0.05) with IC_50_ value of 3.91 ± 0.19*μ*g/mL when compared to the tacrine (IC_50_= 3.50 ± 0.17 *μ*g/mL) used as a standard of AChE inhibition. The n-butanol (IC_50_= 9.97 ± 0.48*μ*g/mL), dichloromethane (IC_50_= 15.78 ± 0.78*μ*g/mL), and ethyl acetate (IC_50_= 17.76 ± 0.88*μ*g/mL) extracts showed moderate AChE inhibitory activity. However, the hexane fraction exhibited no inhibition, and its activity was same as that of control. These results reveal that the active fractions were those obtained by extraction with polar solvents (aqueous and n-butanol). In comparison with other studies, the aqueous extract of* V. lippii* exhibited AChE inhibitory activity higher than that obtained from aqueous extracts of some plants from Argentina [30] and some* Centaurea* species such as* C. antalyense*,* C. polypodiifolia var. pseudobehen,* and* C. pyrrhoblephara *[31].

AChE inhibitors have been extensively used in the treatment of mild to moderate Alzheimer disease. There are several researchers who focused on the quest of new AChE inhibitors from the herbal resources to replace synthetic drugs such as donepezil and tacrine having any adverse effects [32]. For this purpose, the obtained results indicate that* V. lippii* could serve as an inhibitor against the cholinesterase enzyme family and used as complement for the treatment of some Alzheimer diseases. The AChE inhibitory activity of* V. lippii *extracts has never been reported before and their anticholinesterase activity could be attributed to their TP and TF contents (Table 1). Indeed, several authors reported previously a strong relationship between anticholinesterase activity and phenolic content of some* Centaurea* species such as* C. depressa*,* C. drabifolia subsp. Detonsa, C. kotschyi var. persica, C. patula, C. pulchella, C. tchihatcheffi, C. triumfettii, *and* C. urvillei subsp. Hayekiana *[31, 33].

### 3.4. *In Vitro* Alpha-Amylase Inhibitory Assay

This assay evaluated the ability of* V. lippii* extracts to inhibit *α*-amylase activity. The *α*-amylase, a digestive enzyme secreted from the pancreas and salivary gland, is engaged in important biological processes such as digestion of carbohydrates, reducing postprandial hyperglycaemia. The inhibition of this digestive enzyme is therefore used as one of the diabetic treatments [23]. As indicated in Table 3, the IC_50_ values of aqueous, n-butanol, ethyl acetate, and dichloromethane fractions were 7.48 ±0.34, 11.07 ±0.56, 13.28 ±0.65, and 16.99 ±0.81*μ*g/mL, respectively, indicating their promising inhibitory activity against the pancreatic *α*-amylase enzyme. It should be noted that acarbose (standard antidiabetic agent) showed more potent inhibition of *α*-amylase (IC_50_ = 5.54 ±0.27*μ*g/mL) than all tested fractions.

Moreover, the appreciable *α*-amylase inhibitory capacities of these fractions might be attributed to their TP values (Table 1). This suggestion was in agreement with previous studies reporting that many phenolic compounds and flavonoids could prevent the activity of carbohydrate-hydrolyzing enzymes, related to their capacity to bind with proteins [25, 29]. Therefore,* V. lippii* can be considered a new natural source able to fight against type 2 diabetes.

### 3.5. Antibacterial Activity Assay

In the present study, the antibacterial activity of* V. lippii* fractions was evaluated through a set of pathogenic bacteria. The relevant results of this assay were depicted in Table 4. The* V. lippii *extracts inhibited the growth of bacterial strains producing a zone diameter of inhibition from 5.0 to 17.6 mm for Gram-negative bacteria and from 5.25 to 15.5 mm for Gram-positive bacteria. The standard antibiotic, penicillin, showed a strong antibacterial inhibition against practically all bacteria strains. Among Gram-negative bacteria, the strongest activity was observed (p<0.05) for aqueous and n-butanol fractions against* Salmonella enterica* (17.6±0.5 mm and 14.0±0.3 mm, respectively). These values are higher (p<0.05) than that of penicillin, which is sensitive to the bacteria. Generally, plant extracts were usually more active against Gram-positive than Gram-negative bacteria [25]. This could be explained by the fact that Gram-positive strains have only a peptidoglycan layer which is not a selective barrier to plant extracts [26]. The antibacterial activity of* V. lippii* could be assigned to the presence of a high concentration of phenolic compounds which were reported in previous works to exhibit a powerful antimicrobial effect [22, 26].

### 3.6. Activities Discrimination of Ethyl Acetate and Water Fractions by Combining Phytochemical Contents within a Multivariate Analysis: Principal Component Analysis

**Figure 1:**
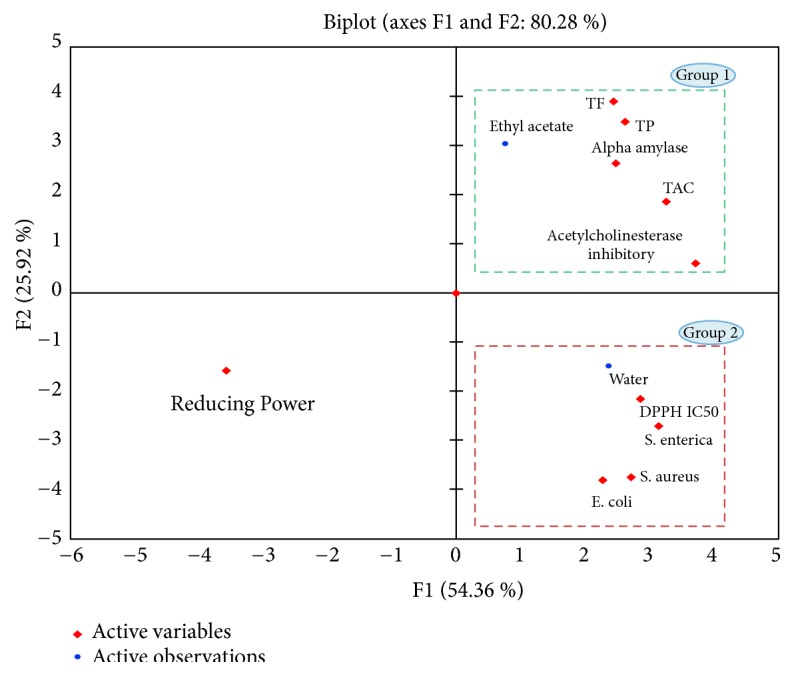
Bioplot representation on the factor-plane (PC1-PC2) showing vector distribution of phytochemical content and activities within score plot of the ethyl acetate and aqueous extracts.

Principal Component Analysis (PCA) was conducted to get a general overview of the data distribution, thus a new set of latent factors or principal components (PCs) was generated. In this part, PCA based on the corresponding data of ethyl acetate and water fractions set including chemical (total phenolic and flavonoid contents), antioxidant (DPPH radical scavenging assay, total antioxidant capacity assay (TAC) and reducing power assay (FRAP)), alpha-amylase inhibitory assay, antimicrobial and acetylcholinesterase enzyme (AChE) inhibitory values of ethyl acetate and water fractions values was carried out (Figure 1).

The first principal component (PC1) had the highest eigenvalue of 5.89 and accounted for 54.36% of the variability in the data set. The second, third, and fourth PCs (PC2, PC3, and PC4) had eigenvalues of 2.851, 1.328, and 0.841 and explained 25.921%, 12.075%, and 7.641% of the variance in the data, respectively. Subsequently, plotting the scores of the samples in the subspaces PC1 vs. PC2 (Figure 1) (80.28% of the total variance of the data) a clear grouping of samples was observable based on solvent extraction (ethyl acetate and water). Moreover, the PCA, which confirms the previous observations, allowed the discrimination of two groups around the PC1 and PC2 axes' components and activities (Figure 1). These axes' components selected positively the group G1 with ethyl acetate fraction correlating with TAC, acetylcholinesterase enzyme (AChE), and alpha-amylase activities. The group G2 mainly constituted by the water fraction which correlate with DPPH radical scavenging and antibacterial (anti-*Salmonella enterica*, anti-*Escherichia coli, *and anti-*Staphylococcus aur*eus) activities.

### 3.7. Qualitative Phytochemical Analyses of Ethyl Acetate and Aqueous Fractions

**Figure 2:**
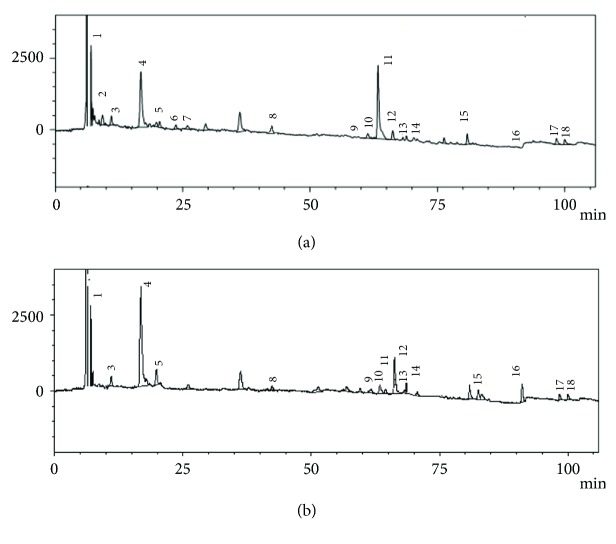
HPLC based peak chromatograms (at 254 nm) of components from ethyl acetate (a) and aqueous (b) extracts of* V. lippii*.

LC-ESI-MS/MS analyses were performed to determine for the first time the chemical profiles of ethyl acetate and aqueous fractions exhibiting remarkable biological activities. The HPLC chromatograms at 254 nm of these two fractions were illustrated in Figure 2.

**Figure 3:**
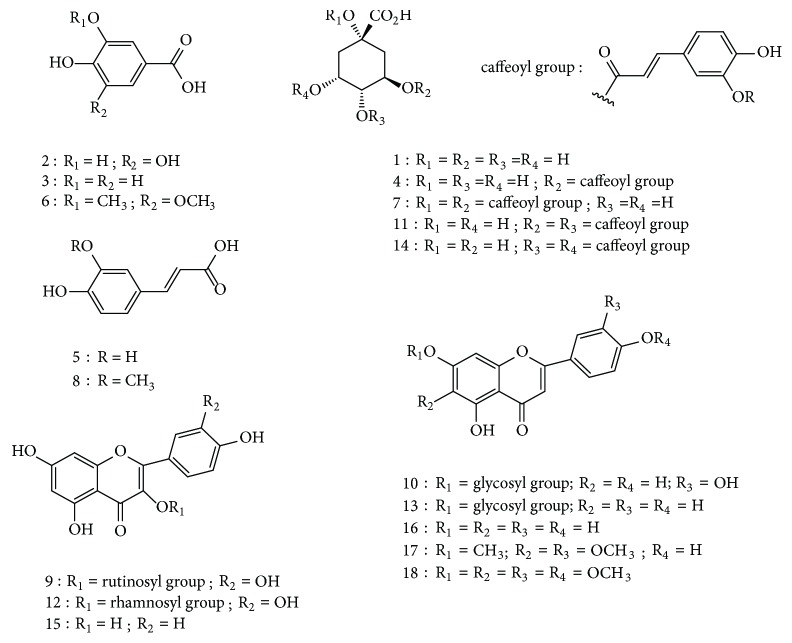
Chemical structures of the acids and flavonoids identified from ethyl acetate and aqueous extracts of* V. lippii*.

Eighteen constituents, numbered 1-18, were detected and tentatively identified as belonging to both phenolic acid and flavonoid groups. The structures of all identified compounds are shown in Figure 3. The identification of the phenolic compounds was carried out by mass spectra, comparison with reference compounds and with literature data.  Table 5 summarized all the identified peaks with retention times (t_R_), UV values (*λ*_max_), pseudomolecular ions, molecular formula, and main fragment ions.

#### 3.7.1. Quinic Acid Derivatives

The quinic acid derivatives exhibited characteristic UV spectra with an absorption maxima at *λ*_max_ around 320-325nm [34]. Peak 1 (t_R_ 6.21 min) was identified as quinic acid by comparison with a standard and also by its pseudomolecular ion [M-H]^−^ at m/z 191 and fragmentation pattern at m/z 173 [quinic acid-H-H_2_O]^−^   [35]. Peak 4 (t_R_ 16.93 min) was identified as 3-*O*-caffeoylquinic acid that was assigned according to the MS^2^ fragment ions at m/z 191 [quinic acid-H]^−^ and at m/z 179 [caffeic acid-H]^−^ and by comparison with an authentic compound [36].

Additionally, three di-*O*-caffeoylquinic acid isomers were characterized by their deprotonated parent ion [M-H]^−^ at m/z 515 and by similar fragmentation patterns at m/z 191 [M-H-caffeoyl-caffeoyl]^−^, 173 [quinic acid-H-H_2_O]^−^, and 353 [M-H-caffeoyl]^−^ characteristics of dicaffeoylquinic acid. However, by comparing the t_R_ of the standard compounds the peaks 7 (t_R_ 26.03 min), 11 (t_R_ 63.49 min), and 14 (t_R_ 70.36 min) were identified as 1,3-di-*O*-caffeoylquinic; 3,4-di-*O*-caffeoylquinic; and 4,5-di-*O*-caffeoylquinic acids, respectively [35, 36]. It should be noted that 1,3-di-*O*-caffeoylquinic acid was only detected in the aqueous fraction.

#### 3.7.2. Phenolic Acid Derivatives

The MS spectra of compounds 2, 3, 5, 6, and 8 indicated specific fragments which prove the presence of free phenolic acids (Table 5). The UV spectra of peaks 5 and 8 showed the same absorption maximum at 332nm typical of cinnamic acid derivatives [34]. Peak 5 (t_R_ 21.22 min) was identified as caffeic acid by comparing with a reference compound and according to its deprotonated molecular ion [M-H]^−^ at m/z 179 and MS^2^ ions at m/z 161 [caffeic acid-H-H_2_O]^−^ and at m/z 135 [caffeic acid-H-CO_2_]^−^ [37, 38]. Ferulic acid (peak 8, t_R_ 42.30 min) was easy to identify by its MS ions at m/z 191 [M-H]^−^ and at m/z 149 [ferulic acid-H-CO_2_]^−^ [37]. Moreover, peaks 2 (t_R_ 7.86 min), 3 (t_R_ 11.21 min), and 6 (t_R_ 23.77 min) were tentatively assigned as gallic, protocatechuic, and syringic acids, respectively, based on the UV spectra, MS^2^ fragmentation pattern and previous report [38]. Gallic and syringic acids were only detected in the aqueous fraction.

#### 3.7.3. Flavonoids

In ethyl acetate and aqueous fractions, flavonoids were represented with kaempferol and quercetin derivatives that exhibited typical UV spectra with band II in the 252-257nm range and band I in the 350-359 nm range [34].

The assignment of peak 15 (t_R_ 80.84 min) as kaempferol was done via its pseudomolecular ion [M-H]^−^ at m/z 285 and MS^2^ ions at m/z 269 and 179 [37]. Compounds 9 (t_R_ 59.01 min) and 12 (t_R_ 67.22 min) produced characteristic MS^2^ ion at m/z 301 which corresponds to quercetin aglycone. Peak 9 revealed parent ion [M-H]^−^ at m/z 609 that releases MS^2^ fragments at m/z 463 [M-H-rhamnosyl]^−^ and 301[M-H-rhamnosyl-glucosyl]^−^; therefore, it was identified as rutin [37]. While peak 12, having MS ions at m/z 447 [M-H]^−^ and 301[M-H-rhamnosyl]^−^, was associated with quercetrin (quercetin-3-*O*-rhamnoside) [39].

Besides flavonols, five flavonones, possessing similar UV spectra, were discerned in the ethyl acetate and aqueous fractions. Apigenin (peak 16, t_R_ 89.16 min) was easy to identify by its MS fragmentation ions at m/z 269 [M-H]^−^ and at m/z 179, 151, and 119 [35]. On the basis of literature data [36], peak 10 (t_R_ 61.03 min) was tentatively assigned as luteolin-7-*O*-glucoside (MS ions at m/z 447[M-H]^−^ and 285 [luteolin-H]^−^). Compounds 13 (t_R_ 68.63 min), 17 (t_R_ 99.73 min), and 18 (t_R_ 102.51 min) were identified as apigenin-7-*O*-glucoside, cirsilineol, and acacetin, respectively, by comparison with standard compounds and with literature data [40].

### 3.8. Quantitative Phytochemical Analyses of Ethyl Acetate and Aqueous Fractions

The quantification analyses data of the identification constituents were achieved by HPLC. The amounts of the compounds, detected in the samples and expressed in *μ*g/g of dry material, were reported in Table 5. The results showed that quinic acid derivatives were the dominant components in ethyl acetate and aqueous fractions with percentages 95.93 and 96.96 %, respectively. On the other hand, phenolic acids and flavonoids were found for the two fractions in lower levels of contents which are less than 200 *μ*g/g of dry material. Quinic, 3-*O*-caffeoylquinic, and 3,4-di-*O*-caffeoylquinic acids were the most abundant acids in the two samples. The major quinic acid derivative in the ethyl acetate fraction was 3,4-di-*O*-caffeoylquinic acid (9849.46 *μ*g/g of dry material) while 3-*O*-caffeoylquinic acid was found the dominant compound in the aqueous fraction (11721.58 *μ*g/g of dry material). Several works reported that the 3,4-di-*O*-caffeoylquinic acid exhibited antioxidant [41], antidiabetic [42], antibacterial [43], cytotoxic [42], and anti-HVS-1 effects [44]. Also, 3-*O*-caffeoylquinic acid was mentioned possessing antioxidant [45], antidiabetic [46], antimicrobial [47], and anti-Alzheimer activities [48]. Thus, the biological activities of* V. lippii* fractions could be related to the high amounts of quinic acid derivatives and specially 3-*O*-caffeoylquinic and 3,4-di-*O*-caffeoylquinic acids.

## 4. Conclusions

This paper is the first report that highlights the antioxidant, antiacetylcholinesterase, antidiabetic, and antibacterial proprieties of* V. lippii*. Among all the extracts, ethyl acetate and aqueous fractions exhibited the greater biological activities and the highest levels in total phenolic and flavonoid contents. LC-ESI-MS/MS analyses of these two fractions led to the identification of eighteen compounds belonging to phenolic acids and flavonoids, where quinic, 3-*O*-caffeoylquinic and 3,4-di-*O*-caffeoylquinic acids were present in higher amounts. Therefore, the qualitative and quantitative analyses of major constituents in* V. lippii* samples could be helpful for understanding the relationship between the total phenolic and flavonoid contents and their biological activities. However, further studies are required to isolate new bioactive components in the ethyl acetate and aqueous fractions and to evaluate their* in vivo* biological capacities.

## Figures and Tables

**Table 1:** Total phenolic (TP) and flavonoid (TF) contents, DPPH radical scavenging, reducing power and total antioxidant capacity (TAC) activities of extracts from *V. lippii*.

Sample	Total phenolic (mg GAE/g extract )	Total flavonoid (mg QE/g extract )	DPPH	TAC (mg vitamin E/g extract)	Reducing power EC_50_ (*μ*g/mL)
			IC_50_ (*μ*g/mL)		
Hexane	7.46±0.23^a^	2.14±0 .05^a^	inactive	45.97±0.05^a^	383.33±2.85^e^
Dichloromethane	24.13±0.04^b^	10.49±0.02^c d^	34.25±1.69^c^	64.37±0.03^b^	216.66±2.49^d^
Ethyl acetate	65.22±0.03^d^	12.50±0.04^d^	11.50±0.57^a^	105.21±0.01^e^	55.40±2.00^b^
n-Butanol	38.83±0.07^c^	5.00±0.03^b^	43.77±2.09^d^	81.68±0.01^c^	150.00±3.12^c^
Aqueous	35.04±0.05^c^	8.90±0.04^c^	28.81±1.35^b^	98.77±0.02^d^	66.65±1.40^b^
BHT	-	-	13.00±0.57^a^	-	43.35±0.95^a^

±: standard deviation of three replicates.

a–e: averages with different letters in the same column are different (P<0.05).

**Table 2:** Acetylcholinesterase inhibitory activity of *V. lippii *extracts.

Sample	IC_50_ (*μ*g/mL)
Hexane	inactive
Dichloromethane	15.78 ± 0.78^c^
Ethyl acetate	17.76 ± 0.88^d^
n-Butanol	9.97 ± 0.48^b^
Aqueous	3.91 ± 0.19^a^
Tacrine	3.50 ± 0.17^a^

±: standard deviation of three replicates.

a–d: averages with different letters in the same column are different (P<0.05).

**Table 3:** Pancreatic *α*-amylase inhibition assay of *V. lippii *extracts.

Sample	Concentration (*μ*g/mL)	% Inhibition	IC_50_ (*μ*g/mL)
Hexane	28.57	44.50±0.08^c^	
	14.28	32.54±0.11^b^	inactive
	7.14	17.67±0.06^a^	

Dichloromethane	28.57	88.23±0.09^c^	
	14.28	46.55±0.03^b^	16.99±0.81^e^
	7.14	32.83±0.08^a^	

Ethyl acetate	28.57	90.02±0.10^c^	
	14.28	62.73±0.13^b^	13.28±0.65^d^
	7.14	38.36±0.09^a^	

n-Butanol	28.57	92.13±0.02^c^	
	14.28	67.44±0.05^b^	11.07±0.56^c^
	7.14	40.54±0.11^a^	

Aqueous	28.57	94.70±0.12^c^	
	14.28	74.13±0.09^b^	7.48±0.34^b^
	7.14	48.16±0.10^a^	

Acarbose	11.42	86.62±0.04^c^	
	5.71	51.52±0.02^b^	5.54±0.27^a^
	2.85	29.80±0.07^a^	

±: standard deviation of three replicates.

a–e: averages with different letters in the same column are different (P<0.05).

**Table 4:** Antibacterial activity of *V. lippii* extracts using agar disc diffusion method.

Sample	*Salmonella enterica*	*Escherichia coli*	*Enterococcus faecalis*	*Staphylococcus aureus *	*Bacillus thuringiensis*
	CIP 8039	ATCC 8739	ATCC29212	ATCC 6538	
Hexane	7.33±0.3^a^	5.25±0.1^a^	5.25±0.1^a^	5.25±0.1^a^	8.33±0.3^b^
Dichloromethane	9.25±0.5^b^	7.8±0.6^b^	5.75±0.2^a^	5.0±0.1^a^	5.83±0.2^a^
Ethyl acetate	10.5±0.4^b^	5.0±0.2^a^	6.33±0.2^a^	5.33±0.1^a^	5.5±0.1^a^
n-Butanol	14.0±0.3^c^	12.0±0.4^c^	5.50±0.1^a^	15.4±0.3^c^	5.0±0.2^a^
Aqueous	17.6±0.5^d^	12.2±0.1^c^	8.40±0.32^b^	10.5±0.6^b^	15.5±0.3^c^
Penicillin	R	16.3±0.1^d^	16.0±0.2^c^	14.1±0.5^c^	18.0±0.4^d^

R= resistant. Inhibition zone in diameter (mm ± SD) around the discs impregnated with 100 *μ*g per disc of each extract. Penicillin (10 *μ*g / disc) was used as positive control for bacteria.

±: standard deviation of three replicates.

a–d: averages with different letters in the same column are different (P<0.05).

**Table 5:** Qualitative and quantitative phytochemical analyses of ethyl acetate and aqueous extracts of *V. lippii*.

Peak	t_R_(min)	*λ*_max_(nm)	[M-H]^−^ (*m/z*)	Main fragment ions MS^2^ (*m*/*z*)	Molecular formula	Tentative identification	Content (*μ*g/g of Dried material)	
							Ethyl acetate extract	Aqueous extract
1	6.21	321	191	173, 135	C_7_H_12_O_6_	Quinic acid^a^	1265.104	7697.052
2	7.86	280, 216	169	125	C_7_H_6_O_5_	Gallic acid	89.940	-
3	11.21	294	153	109	C_7_H_6_O_4_	Protocatechuic acid^a^	136.743	89.051
4	16.93	324, 218	353	191, 179	C_16_H_18_O_9_	Chlorogenic acid^a^	5868.611	11721.583
5	21.22	332, 270	179	161, 135, 143	C_9_H_8_O_4_	Caffeic acid^a^	57.846	55.191
6	23.77	280, 210	197	182, 167, 153,138	C_7_H_12_O_6_	Syringic acid	106.743	-
7	26.03	325	515	353, 191, 173	C_16_H_17_O_9_	1,3-di-*O*-caffeoylquinic acid^a^	318.634	-
8	42.30	332, 234	193	178, 149, 134	C_10_H_9_O_4_	Trans ferulic acid	61.567	55.684
9	59.01	355, 254	609	463, 301, 271	C_27_H_30_O_16_	Rutin^a^	38.684	41.863
10	61.03	338, 270	447	285	C_21_H_20_O_11_	Luteolin-7-*O*-glucoside	115.247	198.499
11	63.49	324	515	353, 191, 173	C_16_H_17_O_9_	3,4-di-*O*-caffeoylquinic acid^a^	9849.464	1504.628
12	67.22	353, 256	447	301	C_21_H_20_O_11_	Quercetrin	53.336	83.025
13	68.63	334, 217	431	269	C_21_H_20_O_10_	Apigenin-7-*O*-glucoside^a^	7.350	10.695
14	70.36	323	515	353, 191, 179, 173	C_16_H_17_O_9_	4,5-di-*O*-caffeoylquinic acid^a^	149.012	101.494
15	80.84	365, 264	285	269, 179	C_15_H_10_O_6_	Kaempferol	56.397	102.209
16	89.16	334, 268	269	179, 151, 119	C_15_H_10_O_6_	Apigenin	2.408	15.739
17	99.73	340, 270	343	297, 255, 241	C_18_H_16_O_7_	Cirsilineol^a^	2.255	0.760
18	102.51	332, 269	283	240, 151, 131	C_16_H_12_O_5_	Acacetin^a^	10.893	7.895
							10,893	7697,052

^a^Identity confirmed by standards; (-): absence in extract.

## Data Availability

All data used to support the findings of this study are included within the article.
